# Elexacaftor/tezacaftor/ivacaftor in children aged ≥6 years with cystic fibrosis heterozygous for *F508del* and a minimal function mutation: results from a 96-week open-label extension study

**DOI:** 10.1183/13993003.02435-2024

**Published:** 2025-07-10

**Authors:** Marcus A. Mall, Claire E. Wainwright, Julian Legg, Mark Chilvers, Sylvia Gartner, Anna-Maria Dittrich, Florian Stehling, Sarah Conner, Sebastian Grant, Nina Suresh, Tanya G. Weinstock, Jane C. Davies, for the VX20-445-119 Study Group

**Affiliations:** 1 Department of Pediatric Respiratory Medicine, Immunology and Critical Care Medicine, Charité – Universitätsmedizin Berlin, Berlin, Germany; 2 German Center for Child and Adolescent Health (DZKJ), partner site, Berlin, Germany; 3 German Center for Lung Research (DZL), associated partner site Berlin, Berlin, Germany; 4 Queensland Children's Hospital, University of Queensland, South Brisbane, Australia; 5 National Institute for Health Research, Southampton Respiratory Biomedical Research Centre, University Hospitals Southampton NHS Foundation Trust, Southampton, UK; 6 Southampton Children's Hospital, University Hospitals Southampton NHS Foundation Trust, Southampton, UK; 7 British Columbia Children's Hospital, University of British Columbia, Vancouver, BC, Canada; 8 Hospital Universitari Vall d'Hebron, Barcelona, Spain; 9 Department for Pediatric Pulmonology, Allergology and Neonatology, Hannover Medical School, Hannover, Germany; 10 BREATH, German Center for Lung Research (DZL), Hannover, Germany; 11 Children's Hospital, University of Duisburg-Essen, Essen, Germany; 12 Vertex Pharmaceuticals Incorporated, Boston, MA, USA; 13 National Heart and Lung Institute, Imperial College London, London, UK; 14 Royal Brompton and Harefield Hospitals, Guy's and St Thomas’ NHS Trust, London, UK; 15 European CF Society Lung Clearance Index Core Facility, London, UK; 16 Co-lead authors

## Abstract

**Background:**

Elexacaftor/tezacaftor/ivacaftor (ELX/TEZ/IVA) was efficacious and safe in children aged 6–11 years with cystic fibrosis (CF) heterozygous for *F508del* and a minimal function CF transmembrane conductance regulator (*CFTR*) variant (*F*/MF genotypes) in a 24-week, placebo-controlled trial. We conducted a 96-week open-label extension study for children who completed the 24-week parent study.

**Methods:**

In this phase 3b extension study, dosing was based on weight and age, with children weighing <30 kg and aged <12 years receiving ELX 100 mg once daily, TEZ 50 mg once daily and IVA 75 mg every 12 h, and children ≥30 kg or ≥12 years receiving ELX 200 mg once daily, TEZ 100 mg once daily and IVA 150 mg every 12 h. The primary end-point was safety and tolerability. Secondary and other efficacy end-points included absolute changes from parent study baseline in sweat chloride concentration, lung clearance index (LCI_2.5_), percentage predicted forced expiratory volume in 1 s (FEV_1_) and Cystic Fibrosis Questionnaire-Revised (CFQ-R) respiratory domain score.

**Results:**

A total of 120 children were enrolled and dosed. 118 children (98.3%) had adverse events (AEs), which for most were mild (43.3%) or moderate (48.3%) in severity. The most common AEs (≥20% of children) were COVID-19 (58.3%), cough (51.7%), nasopharyngitis (45.0%), pyrexia (40.0%), headache (37.5%), upper respiratory tract infection (30.8%), oropharyngeal pain (26.7%), rhinitis (24.2%), abdominal pain (22.5%) and vomiting (20.0%). Children who transitioned from the placebo and ELX/TEZ/IVA groups of the parent study had improvements from parent study baseline at Week 96 in mean sweat chloride concentration (−57.3 (95% CI −61.6– −52.9) and −57.5 (95% CI −62.0– −53.0) mmol·L^−1^), LCI_2.5_ (−1.74 (95% CI −2.09– −1.38) and −2.35 (95% CI −2.72– −1.97) units), FEV_1_ % pred (6.1 (95% CI 2.6–9.7) and 6.9 (95% CI 3.2–10.5) percentage points) and CFQ-R respiratory domain score (6.6 (95% CI 2.5–10.8) and 2.6 (95% CI −1.6–6.8) points).

**Conclusions:**

ELX/TEZ/IVA treatment was generally safe and well tolerated, with a safety profile consistent with the parent study and older age groups. After starting ELX/TEZ/IVA, children had robust improvements in sweat chloride concentration and lung function that were maintained through 96 weeks. These results demonstrate the safety and durable efficacy of ELX/TEZ/IVA in this paediatric population.

## Shareable abstract

**In this 96-week open-label extension study in children with CF aged ≥6 years with *F*/MF genotypes, ELX/TEZ/IVA treatment remained generally safe and well tolerated, and led to sustained improvements in lung function, CFTR function and respiratory symptoms** https://bit.ly/3DP9tXX

## Graphical abstract

**Figure:**
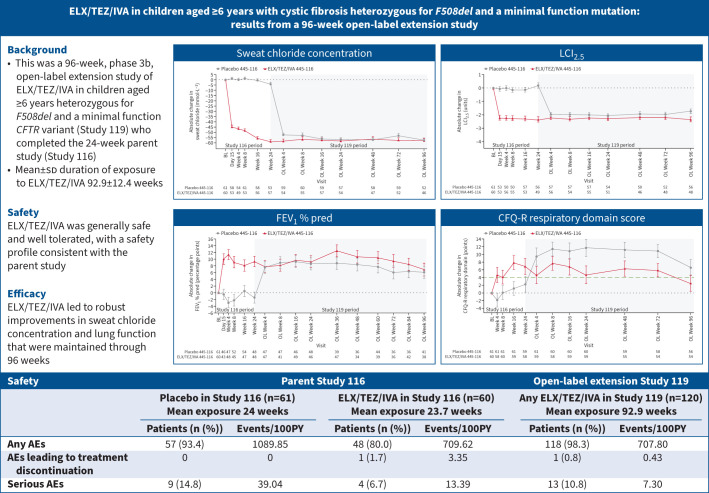
Overview of the study. ELX: elexacaftor; TEZ: tezacaftor; IVA: ivacaftor; *CFTR*: cystic fibrosis transmembrane conductance regulator; BL: baseline; OL: open-label; LCI_2.5_: lung clearance index; FEV_1_: forced expiratory volume in 1 s; CFQ-R: Cystic Fibrosis Questionnaire-Revised (minimal clinically important difference shown as a green dashed line); AE: adverse event; events/100PY: number of events per 100 patient-years (336 days=48 weeks per year)=number of events/total duration of treatment-emergent period for each study in 100PY. Data in the graphs are presented as least squares mean±se.

## Introduction

Cystic fibrosis (CF) is an autosomal recessive disease affecting more than 105 000 people worldwide [1–3]. CF is the result of pathogenic variants in the CF transmembrane conductance regulator (*CFTR*) gene leading to mucus buildup in the lungs, pancreas and other secretory organs due to impairment of ion and water transport across cell membranes [1, 4]. Early diagnosis and treatment interventions can limit organ damage and improve long-term outcomes for children with CF [2, 5–7].

Ivacaftor (IVA) is a small-molecule therapeutic designed to improve the flow of chloride by improving CFTR gating [8, 9], while tezacaftor (TEZ) and elexacaftor (ELX) facilitate trafficking of CFTR proteins to the cell membrane thereby increasing cell surface expression of CFTR proteins [10, 11]. In pivotal phase 3 trials in adolescents and adults with CF ≥12 years of age with at least one *F508del* allele, the triple combination ELX/TEZ/IVA was shown to be safe and effective, improving lung function, respiratory symptoms and CFTR function [12, 13]. Globally, *F508del* is the most commonly occurring *CFTR* pathogenic variant amongst people with CF of Northern European ancestry [14]. Although the prevalence of this variant is lower in other populations, it is estimated that ELX/TEZ/IVA has the potential to treat ∼70–90% of patients with CF [14, 15].

Given the importance of early intervention in changing the trajectory of CF disease, an open-label phase 3 study (VX18-445-106) was conducted to assess the safety, pharmacokinetics and efficacy of ELX/TEZ/IVA in children aged 6–11 years heterozygous for *F508del* and a minimal function *CFTR* variant (*F*/MF) or homozygous for *F508del* (*F/F*). That study showed that, consistent with trials in adolescents and adults, ELX/TEZ/IVA was safe in this paediatric group and led to improvements in percentage predicted forced expiratory volume in 1 s (FEV_1_), Cystic Fibrosis Questionnaire-Revised (CFQ-R) respiratory domain score, lung clearance index (LCI_2.5_) and sweat chloride concentration [16]. To further elucidate the impact of ELX/TEZ/IVA in this paediatric population, a 24-week placebo-controlled phase 3b trial was conducted to assess the efficacy and safety in children with *F*/MF genotypes. Significant improvements in lung function along with robust improvements in respiratory symptoms and CFTR function were reported [17]. These results suggest that, consistent with adolescents and adults, ELX/TEZ/IVA has the potential to change CF disease trajectory in children.

Children who completed the 24-week placebo-controlled trial were given the opportunity to enrol in a 96-week extension study to assess the safety and efficacy of ELX/TEZ/IVA. Here we report the results from this open-label extension study. Some results were previously reported in a conference abstract (Deutschen Mukoviszidose Tagung (DMT), 23–25 November 2023, Wurzburg, Germany).

## Methods

### Patients, trial design and oversight

This study (VX20-445-119 (Study 119); ClinicalTrials.gov: NCT04545515; EudraCT: 2020-001404-42) was a phase 3b, multicentre, 96-week open-label extension study for children aged ≥6 years with CF, heterozygous for *F508del* and a minimal function *CFTR* variant (*F*/MF) who completed the 24-week parent study (VX19-445-116 (Study 116) for children aged 6–11 years; a phase 3b, randomised, double-blind, placebo-controlled, multicentre trial) and met the eligibility criteria. The definition of a minimal function variant along with a list of qualifying minimal function variants can be found in the supplementary methods and supplementary table S1. A schematic of the study design is shown in supplementary figure S1.

Dosing was based on age and weight: children aged ≥6– <12 years weighing <30 kg at study entry received ELX 100 mg once daily, TEZ 50 mg once daily and IVA 75 mg every 12 h, while children weighing ≥30 kg or aged ≥12 years received ELX 200 mg once daily, TEZ 100 mg once daily and IVA 150 mg every 12 h (adult dose).

In collaboration with the authors, the trial was designed by Vertex Pharmaceuticals Incorporated. Written informed consent was obtained from the child's legal representative/guardian and assent obtained per local guidelines. Safety was monitored by an independent data safety monitoring committee. Vertex Pharmaceuticals Incorporated performed data collection and analysis. All authors had complete access to the data, reviewed the manuscript and approved it for submission. The study investigators assure accuracy and completeness of the data, and the investigators and Vertex Pharmaceuticals Incorporated assure the fidelity of the trial to the study protocol.

Because parts of this trial overlapped with the severe acute respiratory syndrome coronavirus 2 (SARS-CoV-2) pandemic, a global protocol addendum was implemented to enable children to continue in the study while ensuring safety by minimising risk to SARS-CoV-2 exposure. Measures were adapted based on country and local regulations and site-level considerations. These measures included shipment of study drug from site to home, telephone/video calls for safety assessments, in-home assessments, use of local laboratories for safety assessments, at-home pregnancy testing, verbal re-consenting, remote re-consenting, remote monitoring/source data verification and electronic data capture visit type forms.

### Outcome measures

The primary end-point was safety and tolerability as assessed by adverse events (AEs), clinical laboratory values, ECG, vital signs and pulse oximetry. Secondary end-points were absolute change in sweat chloride concentration and standard LCI at 2.5% of the starting gas concentration (LCI_2.5_) from parent study baseline. Nitrogen multiple-breath washout testing was performed with an Exhalyzer D using Spiroware version 3.1.6 (Eco Medics, Duernten, Switzerland). This version of Spiroware has since been updated to correct for cross-sensitivity issues associated with the oxygen and carbon dioxide sensors in the device that can cause overestimation of nitrogen concentrations [18]. A study by Robinson *et al*. [19] that reassessed 1036 previous tests using the updated software found that although the corrected algorithm did result in lower LCI_2.5_ values, the interpretations and significance of observed treatment effects did not change. Other end-points were absolute change in FEV_1_ % pred and CFQ-R respiratory domain score from parent baseline.

### Statistical analysis

Safety and efficacy analyses included all participants who received at least one dose of study drug. Analysis of safety data was descriptive, and there was no statistical testing. Secondary and other efficacy end-points (absolute change in sweat chloride concentration, LCI_2.5_, FEV_1_ % pred and CFQ-R respiratory domain score from baseline) were analysed using a mixed effects model for repeated measures with absolute change from baseline at each post-baseline visit as the dependent variable. The model included parent study treatment group, visit and parent study treatment-by-visit interaction as fixed effects with continuous parent study baseline LCI_2.5_ and weight at screening visit of parent study (<30 *versus* ≥30 kg) as covariates. The least squares (LS) mean changes from parent study baseline and two-sided 95% confidence intervals at each visit were estimated for each treatment group.

## Results

### Population

The trial took place at 34 sites in Australia, Canada, Denmark, France, Germany, Israel, the Netherlands, Spain, Switzerland and the UK between 11 January 2021 and 24 March 2023. A total of 120 children were enrolled and received at least one dose of study drug. Demographics and clinical characteristics at parent study baseline are presented in table 1.

**TABLE 1:** Demographic and clinical characteristics at parent study baseline^#^

	Placebo in Study 116 (n=61)	ELX/TEZ/IVA in Study 116 (n=59)	Study 119 (n=120)
**Female**	35 (57.4)	34 (57.6)	69 (57.5)
**Age at baseline (years)**	9.2±1.7	9.0±1.8	9.1±1.7
**Race**			
White	42 (68.9)	45 (76.3)	87 (72.5)
Black or African American	0	1 (1.7)	1 (0.8)
Asian	0	1 (1.7)	1 (0.8)
American Indian or Alaska Native	0	1 (1.7)	1 (0.8)
Native Hawaiian or Other Pacific Islander	0	0	0
Other	1 (1.6)	0	1 (0.8)
Not collected per local regulations	18 (29.5)	10 (16.9)	28 (23.3)
Multiracial	0	1 (1.7)	1 (0.8)
**Ethnicity**			
Hispanic or Latino	0	1 (1.7)	1 (0.8)
Not Hispanic or Latino	42 (68.9)	48 (81.4)	90 (75.0)
Not collected per local regulations	19 (31.1)	10 (16.9)	29 (24.2)
**Sweat chloride (mmol·L^−1^)**	102.6±8.6	102.8±10.0	102.7±9.3
**LCI_2.5_ (units)**	9.75±1.95	10.21±2.20	9.98±2.08
**FEV_1_ % pred category**			
<70% pred	10 (16.4)	4 (6.8)	14 (11.7)
≥70– ≤90% pred	23 (37.7)	19 (32.2)	42 (35.0)
>90% pred	28 (45.9)	36 (61.0)	64 (53.3)
**FEV_1_ % pred at baseline**	87.2±15.8	91.6±13.8	89.4±15.0
**CFQ-R respiratory domain score (Child version) (points)**	82.7±14.1	86.0±11.5	84.3±13.0

Data are presented as n (%) or mean±sd. ELX: elexacaftor; TEZ: tezacaftor; IVA: ivacaftor; LCI_2.5_: lung clearance index; FEV_1_: forced expiratory volume in 1 s; CFQ-R: Cystic Fibrosis Questionnaire-Revised. ^#^: baseline was defined as the most recent non-missing measurement before the first dose of study drug in the treatment period of the parent study.

**FIGURE 1:**
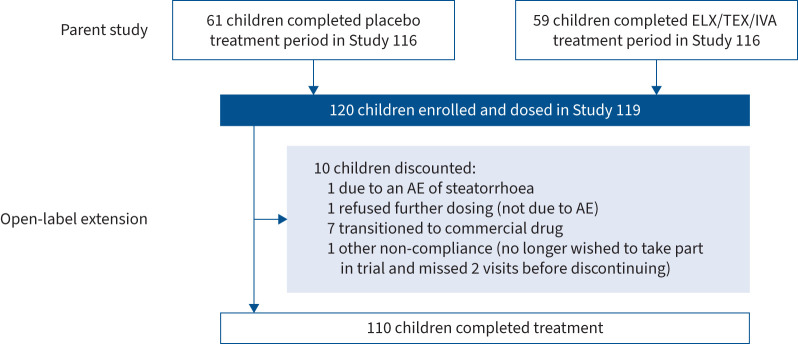
Participant disposition. ELX: elexacaftor; TEZ: tezacaftor; IVA: ivacaftor; AE: adverse event.

The mean±sd exposure to ELX/TEZ/IVA in this extension study was 92.9±12.4 weeks (supplementary table S2), representing 232.2 patient-years of exposure. 10 children discontinued the study (adverse event (AE) of steatorrhoea (n=1), refused further dosing not due to AE (n=1), transitioned to commercial drug (n=7) or other due to non-compliance (n=1)) (figure 1).

### Safety

118 (98.3%) children had at least one AE (table 2). The majority of children experienced AEs that were mild (43.3%) or moderate (48.3%) in severity. 13 (10.8%) children had at least one serious AE (SAE). The only SAE reported in more than one child was infective pulmonary exacerbation of CF which occurred in two children (1.7%) (supplementary table S4). One child (0.8%) discontinued study drug due to a SAE of steatorrhoea that subsequently resolved and was assessed as related to study drug. Alanine aminotransferase (ALT) or aspartate aminotransferase (AST) >3× and >5× upper limit of normal (ULN) occurred in 11 (9.2%) and six (5.0%) children, respectively; no children had ALT or AST >8×ULN (supplementary table S5). There were no children who had ALT or AST >3×ULN concurrent with total bilirubin elevation >2×ULN. Overall, 11 children (9.2%) had at least one elevated transaminase event (supplementary table S6). All elevated transaminase events were mild or moderate in severity, and none were serious. Rash events occurred in 13 children (10.8%), all of which were mild or moderate in severity, and none were serious (supplementary table S7). Overall, 10 out of 69 female children (14.5%) and three out of 51 male children (5.9%) had rash events. Of the 13 children who had rash events in this extension study, 10 had a single rash event and three had more than one rash event. There were five children who had a rash event within 1 month of starting ELX/TEZ/IVA, two of which were considered not related or unlikely to be related to ELX/TEZ/IVA. There were no trends observed in creatine kinase concentration levels, haematology parameters, coagulation, urinalysis, ECG or vital signs. Changes in blood pressure remained stable across the parent and open-label extension studies (supplementary table S8). Three children (2.5%) had an AE of cataract and one child (0.8%) had an AE of lenticular opacities, all of which were mild and non-serious, did not lead to change in study drug dosing, and were not considered clinically significant by investigators. None of the children had a history of lens opacity or cataract, or had any relevant findings at the baseline ophthalmological exam. All events of cataracts were ongoing at the time of study completion. Overall, AEs were generally consistent with common manifestations of CF disease or with common illnesses in children with CF aged ≥6 years. The exposure-adjusted AE rates in the open-label extension study were similar to the active arm of the parent study, and lower than the placebo arm of the parent study (table 2).

**TABLE 2:** Adverse events (AEs)

	Parent Study 116				Open-label extension Study 119	
	Placebo in Study 116 (n=61) Mean exposure 24 weeks		ELX/TEZ/IVA in Study 116 (n=60) Mean exposure 23.7 weeks		Any ELX/TEZ/IVA in Study 119 (n=120) Mean exposure 92.9 weeks	
	n (%)^#^	Events/100PY	n (%)^#^	Events/100PY	n (%)^#^	Events/100PY
**Any AEs**	57 (93.4)	1089.85	48 (80.0)	709.62	118 (98.3)	707.80
**AEs by maximum severity**						
Mild	26 (42.6)	NA	30 (50.0)	NA	52 (43.3)	NA
Moderate	29 (47.5)	NA	16 (26.7)	NA	58 (48.3)	NA
Severe	2 (3.3)	NA	2 (3.3)	NA	8 (6.7)	NA
Life-threatening AEs	0	NA	0	NA	0	NA
**AEs leading to treatment discontinuation**	0	0	1 (1.7)	3.35	1 (0.8)	0.43
**AEs leading to treatment interruption**	0	0	7 (11.7)	33.47	10 (8.3)	6.01
**Serious AEs**	9 (14.8)	39.04	4 (6.7)	13.39	13 (10.8)	7.30
**AEs occurring in at least 20% of children (PT) in Study 119**						
COVID-19	NA	NA	NA	NA	70 (58.3)	33.50
Cough	26 (42.6)	130.13	14 (23.3)	56.90	62 (51.7)	70.01
Nasopharyngitis	9 (14.8)	39.04	7 (11.7)	26.78	54 (45.0)	49.39
Pyrexia	3 (4.9)	9.76	1 (1.7)	3.35	48 (40.0)	36.51
Headache	12 (19.7)	65.07	18 (30.0)	73.64	45 (37.5)	45.53
Upper respiratory tract infection	5 (8.2)	26.03	3 (5.0)	13.39	37 (30.8)	27.92
Oropharyngeal pain	12 (19.7)	45.55	3 (5.0)	13.39	32 (26.7)	21.90
Rhinitis	5 (8.2)	16.27	3 (5.0)	10.04	29 (24.2)	21.47
Abdominal pain	17 (27.9)	87.84	5 (8.3)	30.13	27 (22.5)	18.90
Vomiting	4 (6.6)	13.01	3 (5.0)	13.39	24 (20.0)	18.04

ELX: elexacaftor; TEZ: tezacaftor; IVA: ivacaftor; events/100PY: number of events per 100 patient-years (336 days=48 weeks per year)=number of events/total duration of treatment-emergent period for each study in 100PY; PT: Preferred Term (MedDRA version 25.1 used for Study 119 and MedDRA 24.0 used for Study 116); NA: not applicable. ^#^: when summarising the number and percentage of patients, a patient with multiple occurrences of the same AE or a continuing AE was counted once.

### Efficacy

**FIGURE 2:**
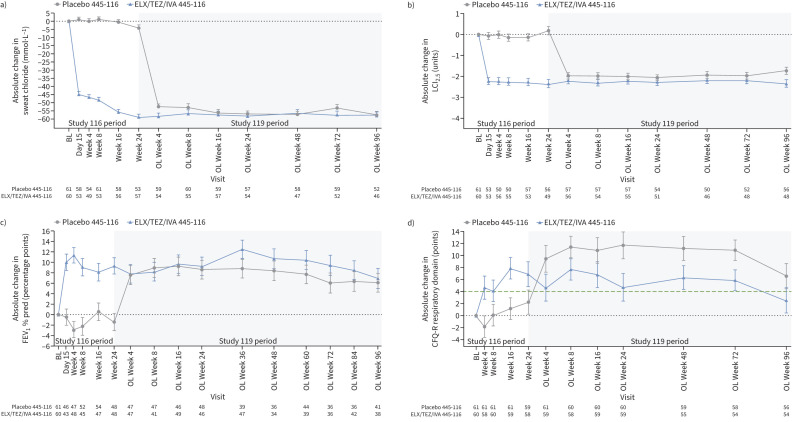
Absolute change from parent study baseline in a) sweat chloride, b) lung clearance index (LCI_2.5_), c) percentage predicted forced expiratory volume in 1 s (FEV_1_) and d) Cystic Fibrosis Questionnaire-Revised (CFQ-R) respiratory domain score at each visit. Parent study baseline was defined as the most recent non-missing measurement before the first dose of study drug in the treatment period of the parent study. Data are presented as least squares mean±se. The minimal clinically important difference for CFQ-R respiratory domain score (4.0 points) is shown as a green dashed line in d). BL: baseline; OL: open-label.

At Week 96, LS mean change in sweat chloride concentration from parent study baseline was −57.3 (95% CI −61.6– −52.9) and −57.5 (95% CI −62.0– −53.0) mmol·L^−1^ for children from the placebo and ELX/TEZ/IVA groups of the parent study, respectively (figure 2a and table 3). The LS mean change in LCI_2.5_ from parent study baseline was −1.74 (95% CI −2.09– −1.38) and −2.35 (95% CI −2.72– −1.97) units for children from the placebo and ELX/TEZ/IVA groups of the parent study, respectively, at Week 96 (figure 2b and table 3). The LS mean change in FEV_1_ % pred from parent study baseline was 6.1 (95% CI 2.6–9.7) and 6.9 (95% CI 3.2–10.5) percentage points for children from the placebo and ELX/TEZ/IVA groups of the parent study, respectively, at Week 96 (figure 2c and table 3). For CFQ-R respiratory domain score, an LS mean change from parent study baseline of 6.6 (95% CI 2.5–10.8) and 2.6 (95% CI −1.6–6.8) points was observed at Week 96 for subjects from the placebo and ELX/TEZ/IVA groups of the parent study, respectively (figure 2d and table 3). LS mean change ranged from 6.6 to 11.7 points for the placebo group of the parent study and from 2.6 to 7.7 points for the ELX/TEZ/IVA group of the parent study. Overall, CFQ-R respiratory domain scores, which were high at baseline, increased following initiation of ELX/TEZ/IVA and were maintained through the 96-week open-label extension period (supplementary figure S2 and supplementary table S3).

**TABLE 3:** Absolute change in sweat chloride, lung clearance index (LCI_2.5_), percentage predicted forced expiratory volume in 1 s (FEV_1_) and Cystic Fibrosis Questionnaire-Revised (CFQ-R) respiratory domain from parent study baseline^#^ at Week 96

	Placebo–ELX/TEZ/IVA (n=61)	ELX/TEZ/IVA–ELX/TEZ/IVA (n=59)
**Absolute change in sweat chloride (mmol·L^−1^) (secondary end-point)**		
n	52	46
LS mean±se	−57.3±2.2	−57.5±2.3
95% CI of LS mean	−61.6– −52.9	−62.0– −53.0
**Absolute change in LCI_2.5_ (units) (secondary end-point)**		
n	56	48
LS mean±se	−1.74±0.18	−2.35±0.19
95% CI of LS mean	−2.09– −1.38	−2.72– −1.97
**Absolute change in FEV_1_ % pred (percentage points) (other end-point)**		
n	41	38
LS mean±se	6.1±1.8	6.9±1.9
95% CI of LS mean	2.6–9.7	3.2–10.5
**Absolute change in CFQ-R respiratory domain score (points) (other end-point)**		
n	56	54
LS mean±se	6.6±2.1	2.6±2.1
95% CI of LS mean	2.5–10.8	−1.6–6.8

ELX: elexacaftor; TEZ: tezacaftor; IVA: ivacaftor; LS: least squares. ^#^: parent study baseline was defined as the most recent non-missing measurement before the first dose of study drug in the treatment period of the parent study.

## Discussion

We evaluated the safety and efficacy of ELX/TEZ/IVA in children with CF aged ≥6 years who had an *F*/MF genotype over a 2-year period. ELX/TEZ/IVA was generally safe and well tolerated, with a safety profile consistent with prior studies [16, 17]. The significant improvements in lung function and robust decreases in sweat chloride concentration previously reported for children given ELX/TEZ/IVA in the parent study were maintained over the period of this extension study. Children who received placebo in the parent study had similar efficacy improvements after starting ELX/TEZ/IVA treatment in this open-label extension study, and these improvements were maintained.

CFTR modulator therapies, such as ELX/TEZ/IVA, require ongoing administration, making studies of long-term safety and efficacy imperative to understanding the clinical benefits of extended use [20]. In the current study, children aged ≥6 years who had previously received ELX/TEZ/IVA in a 24-week, phase 3b, randomised clinical trial, received ELX/TEZ/IVA for an additional 96 weeks. The majority of AEs observed were mild or moderate in severity, and generally consistent with common manifestations of CF in this age group. There was a higher incidence of AEs related to respiratory and upper respiratory tract infection in this extension study than reported in the parent study, most likely the result of symptomatic COVID-19 infections, which were not reported in the parent study that was completed during the early part of the COVID-19 pandemic, but which were the most common AEs in this study (58.3%). Overall, the exposure-adjusted rate of AEs was lower in this extension study than in the parent study. There was a low rate of SAEs (10.8%) and, similar to other AEs, SAEs were generally consistent with common manifestations of CF and complications for children in this age group (*e.g.* COVID-19, cough, nasopharyngitis, pyrexia, headache, upper respiratory tract infection, oropharyngeal pain, rhinitis, abdominal pain and vomiting). One child experienced a SAE of steatorrhoea that was assessed by the study investigators as being related to study drug and resolved after study drug discontinuation. The incidence of transaminase events (9.2%) and rash events (10.8%) was consistent with the parent study and with previous studies of ELX/TEZ/IVA in children aged 6–11 years. Overall, ELX/TEZ/IVA treatment for an additional 96 weeks remained safe and well tolerated, with no new safety findings.

Sweat chloride concentration is an established biomarker of CFTR function, with decreases in sweat chloride concentration suggestive of improved CFTR function in patients with CF [21]. Previous studies, including the parent study of this extension study, showed initiation of ELX/TEZ/IVA treatment led to rapid and robust decreases in sweat chloride concentrations in children aged 6–11 years with CF [16, 17]. Consistent with these previous observations, a robust decrease in mean sweat chloride concentration was seen in children who initiated ELX/TEZ/IVA in this extension study after previously receiving placebo in the parent study. The decreases in sweat chloride concentration following initiation of ELX/TEZ/IVA in both treatment groups were maintained throughout the 96-week treatment period of this extension study. These results suggest that ELX/TEZ/IVA leads to rapid and durable improvements in CFTR function in children with CF.

Significant improvements in lung function and respiratory symptoms were also previously reported in children treated with ELX/TEZ/IVA [17]. While spirometry is typically used to detect impairment in lung function, values for FEV_1_ % pred are often close to the normal range in younger children with CF despite evidence of early structural lung damage [22]. In the current study, robust increases in mean FEV_1_ % pred were seen in children who began ELX/TEZ/IVA treatment in this extension study, consistent with the increases in children given ELX/TEZ/IVA in the parent study, and were maintained through this extension study. To further elucidate the impact of ELX/TEZ/IVA treatment on lung function in this paediatric population, changes in LCI_2.5_ were also assessed. Measures of LCI_2.5_ can provide a more sensitive means to detect early changes in lung function and are considered an appropriate efficacy measurement for younger children with airway diseases [23, 24]. A natural history study reported that children 3–18 years of age with CF not treated with a CFTR modulator had an unadjusted mean annual increase in LCI_2.5_ of 0.29 units [25], as would be expected in a disease characterised by progressive loss of lung function. In contrast, robust decreases in mean LCI_2.5_ were observed in children after beginning treatment with ELX/TEZ/IVA. The improvements in mean LCI_2.5_ seen in both groups after starting ELX/TEZ/IVA were maintained through the 96-week treatment period. Children also had improvements in their respiratory symptoms following the initiation of ELX/TEZ/IVA, with increases in CFQ-R respiratory domain scores. The mean changes from parent study baseline in CFQ-R respiratory domain score ranged from 6.6 to 11.7 points in children who received placebo in the parent study and 2.6 to 7.7 points in those who received ELX/TEZ/IVA in the parent study. The differences in change from baseline between the two treatment groups following start of ELX/TEZ/IVA are likely attributable to a ceiling effect, with smaller changes from baseline observed in the group who previously received ELX/TEZ/IVA in the parent study and had a higher baseline mean CFQ-R respiratory domain score (85.7 *versus* 82.7 points). Despite variability in the change from baseline in mean CFQ-R respiratory domain score at study visits, and a mean change at Week 96 of 2.6 points in children who previously received ELX/TEZ/IVA which was below the established minimal clinically important difference in people with CF and stable disease of 4 points [26], both groups generally maintained improvements in CFQ-R respiratory domain score throughout the extension study and had similar scores at Week 96 (89.6 and 89.2 points) that were in the normal range. Taken together, these results demonstrate that ELX/TEZ/IVA leads to durable improvements in lung function and respiratory symptoms in children with CF.

It should be noted that as this was an open-label study, the lack of a direct comparator group does limit the ability to interpret the safety and efficacy data.

ELX/TEZ/IVA was generally safe and well tolerated for up to 96 weeks of treatment in children ≥6 years, with a safety profile generally consistent with the established safety profile of ELX/TEZ/IVA. The sustained improvements in lung function, respiratory symptoms and CFTR function seen in the parent study were maintained during this open-label extension study for children who received ELX/TEZ/IVA in the parent study. Similarly, children who received placebo in the parent study had efficacy improvements upon initiating ELX/TEZ/IVA that were maintained throughout the open-label extension treatment period. These results confirm the favourable safety profile and durable clinical benefits of ELX/TEZ/IVA in this paediatric population.

## Shareable PDF

10.1183/13993003.02435-2024.Shareable1 This PDF extract can be shared freely online.
Shareable PDF

ERJ-02435-2024.Shareable

## Data Availability

Details on Vertex data sharing criteria and process for requesting access can be found at: www.vrtx.com/independent-research/clinical-trial-data-sharing

## Supplementary material

**Please note:** supplementary material is not edited by the Editorial Office, and is uploaded as it has been supplied by the author. Supplementary material ERJ-02435-2024.Supplement
