# Supplementary material for: Elexacaftor/tezacaftor/ivacaftor in children aged ≥6 years with cystic fibrosis heterozygous for F508del and a minimal function mutation: results from a 96-week open-label extension study
Source: Eur Respir J. 2025 Jul 10;66(1):2402435. doi: 10.1183/13993003.02435-2024 (PMC12256806; doi:10.1183/13993003.02435-2024)
Supplement: Supplementary file 1 [file ERJ-02435-2024.Shareable.pdf]

EUROPEAN RESPIRATORY JOURNAL
ORIGINAL RESEARCH ARTICLE
M.A. MALL ET AL.

SHAREABLE PDF

Elexacaftor/tezacaftor/ivacaftor in children aged ⩾6 years with cystic fibrosis heterozygous for *F508del* and a minimal function mutation: results from a 96-week open-label extension study

Marcus A. Mall, Claire E. Wainwright, Julian Legg, Mark Chilvers, Sylvia Gartner, Anna-Maria Dittrich, Florian Stehling, Sarah Conner, Sebastian Grant, Nina Suresh, Tanya G. Weinstock and Jane C. Davies for the VX20-445-119 Study Group

ELX/TEZ/IVA in children aged ≥6 years with cystic fibrosis heterozygous for *F508del* and a minimal function mutation: results from a 96-week open-label extension study

Background

- This was a 96-week, phase 3b, open-label extension study of ELX/TEZ/IVA in children aged ≥6 years heterozygous for *F508del* and a minimal function *CFTR* variant (Study 119) who completed the 24-week parent study (Study 116)
- Mean±SD duration of exposure to ELX/TEZ/IVA 92.9±12.4 weeks

Safety

ELX/TEZ/IVA was generally safe and well tolerated, with a safety profile consistent with the parent study

Efficacy

ELX/TEZ/IVA led to robust improvements in sweat chloride concentration and lung function that were maintained through 96 weeks

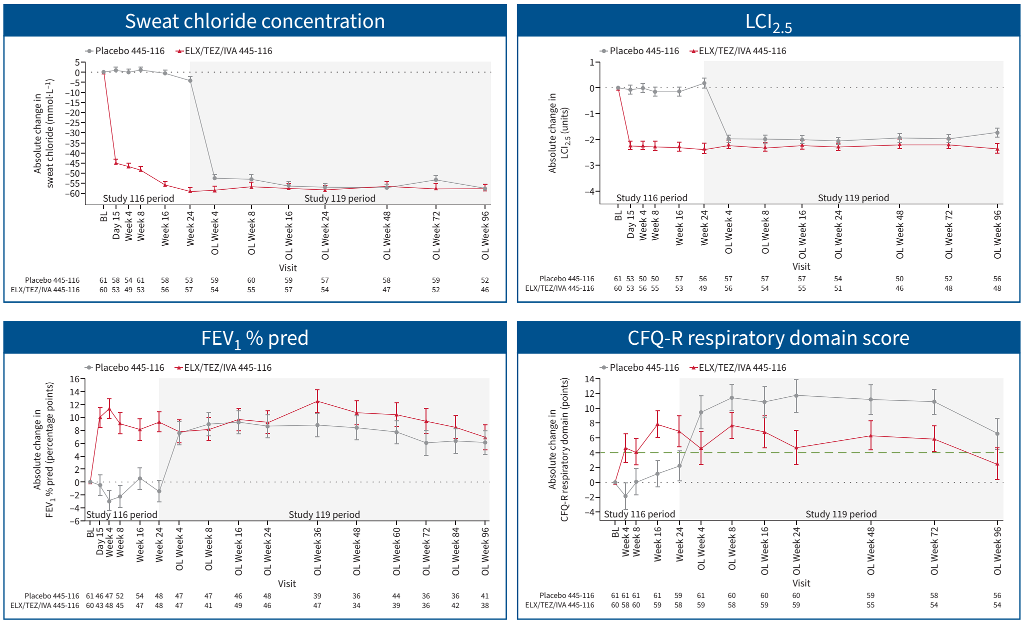

Safety	Parent Study 116				Open-label extension Study 119	
	Placebo in Study 116 (n=61) Mean exposure 24 weeks		ELX/TEZ/IVA in Study 116 (n=60) Mean exposure 23.7 weeks		Any ELX/TEZ/IVA in Study 119 (n=120) Mean exposure 92.9 weeks	
	Patients (n (%))	Events/100PY	Patients (n (%))	Events/100PY	Patients (n (%))	Events/100PY
**Any AEs**	57 (93.4)	1089.85	48 (80.0)	709.62	118 (98.3)	707.80
**AEs leading to treatment discontinuation**	0	0	1 (1.7)	3.35	1 (0.8)	0.43
**Serious AEs**	9 (14.8)	39.04	4 (6.7)	13.39	13 (10.8)	7.30

GRAPHICAL ABSTRACT Overview of the study. ELX: elexacaftor; TEZ: tezacaftor; IVA: ivacaftor; *CFTR*: cystic fibrosis transmembrane conductance regulator; BL: baseline; OL: open-label; $LCI_{2.5}$: lung clearance index; FEV_1: forced expiratory volume in 1 s; CFQ-R: Cystic Fibrosis Questionnaire-Revised (minimal clinically important difference shown as a green dashed line); AE: adverse event; events/100PY: number of events per 100 patient-years (336 days=48 weeks per year)=number of events/total duration of treatment-emergent period for each study in 100PY. Data in the graphs are presented as least squares mean±SE.

SHAREABLE PDF

Elexacaftor/tezacaftor/ivacaftor in children aged ⩾6 years with cystic fibrosis heterozygous for *F508del* and a minimal function mutation: results from a 96-week open-label extension study

Marcus A. Mall[1,2,3,16], Claire E. Wainwright[4,16], Julian Legg[5,6], Mark Chilvers[7], Sylvia Gartner[8], Anna-Maria Dittrich[9,10], Florian Stehling[11], Sarah Conner[12], Sebastian Grant[12], Nina Suresh[12], Tanya G. Weinstock[12] and Jane C. Davies[13,14,15] for the VX20-445-119 Study Group

[1]Department of Pediatric Respiratory Medicine, Immunology and Critical Care Medicine, Charité – Universitätsmedizin Berlin, Berlin, Germany. [2]German Center for Child and Adolescent Health (DZKJ), partner site, Berlin, Germany. [3]German Center for Lung Research (DZL), associated partner site Berlin, Berlin, Germany. [4]Queensland Children's Hospital, University of Queensland, South Brisbane, Australia. [5]National Institute for Health Research, Southampton Respiratory Biomedical Research Centre, University Hospitals Southampton NHS Foundation Trust, Southampton, UK. [6]Southampton Children's Hospital, University Hospitals Southampton NHS Foundation Trust, Southampton, UK. [7]British Columbia Children's Hospital, University of British Columbia, Vancouver, BC, Canada. [8]Hospital Universitari Vall d'Hebron, Barcelona, Spain. [9]Department for Pediatric Pulmonology, Allergology and Neonatology, Hannover Medical School, Hannover, Germany. [10]BREATH, German Center for Lung Research (DZL), Hannover, Germany. [11]Children's Hospital, University of Duisburg-Essen, Essen, Germany. [12]Vertex Pharmaceuticals Incorporated, Boston, MA, USA. [13]National Heart and Lung Institute, Imperial College London, London, UK. [14]Royal Brompton and Harefield Hospitals, Guy's and St Thomas' NHS Trust, London, UK. [15]European CF Society Lung Clearance Index Core Facility, London, UK. [16]Co-lead authors.

Corresponding author: Jane C. Davies (j.c.davies@imperial.ac.uk)

Check for updates

Shareable abstract (@ERSpublications)
In this 96-week open-label extension study in children with CF aged ⩾6 years with *F*/MF genotypes, ELX/TEZ/IVA treatment remained generally safe and well tolerated, and led to sustained improvements in lung function, CFTR function and respiratory symptoms https://bit.ly/3DP9tXX

Cite this article as: Mall MA, Wainwright CE, Legg J, *et al.* Elexacaftor/tezacaftor/ivacaftor in children aged ⩾6 years with cystic fibrosis heterozygous for *F508del* and a minimal function mutation: results from a 96-week open-label extension study. *Eur Respir J* 2025; 66: 2402435 [DOI: 10.1183/13993003.02435-2024].

This PDF extract can be shared freely online.

Copyright ©The authors 2025.

This version is distributed under the terms of the Creative Commons Attribution Licence 4.0.

This article has an editorial commentary: https://doi.org/10.1183/13993003.00793-2025

Received: 13 Dec 2024
Accepted: 10 March 2025

Abstract

Background Elexacaftor/tezacaftor/ivacaftor (ELX/TEZ/IVA) was efficacious and safe in children aged 6–11 years with cystic fibrosis (CF) heterozygous for *F508del* and a minimal function CF transmembrane conductance regulator (*CFTR*) variant (*F*/MF genotypes) in a 24-week, placebo-controlled trial. We conducted a 96-week open-label extension study for children who completed the 24-week parent study.

Methods In this phase 3b extension study, dosing was based on weight and age, with children weighing <30 kg and aged <12 years receiving ELX 100 mg once daily, TEZ 50 mg once daily and IVA 75 mg every 12 h, and children ⩾30 kg or ⩾12 years receiving ELX 200 mg once daily, TEZ 100 mg once daily and IVA 150 mg every 12 h. The primary end-point was safety and tolerability. Secondary and other efficacy end-points included absolute changes from parent study baseline in sweat chloride concentration, lung clearance index ($LCI_{2.5}$), percentage predicted forced expiratory volume in 1 s (FEV_1) and Cystic Fibrosis Questionnaire-Revised (CFQ-R) respiratory domain score.

Results A total of 120 children were enrolled and dosed. 118 children (98.3%) had adverse events (AEs), which for most were mild (43.3%) or moderate (48.3%) in severity. The most common AEs (⩾20% of children) were COVID-19 (58.3%), cough (51.7%), nasopharyngitis (45.0%), pyrexia (40.0%), headache (37.5%), upper respiratory tract infection (30.8%), oropharyngeal pain (26.7%), rhinitis (24.2%), abdominal pain (22.5%) and vomiting (20.0%). Children who transitioned from the placebo and ELX/TEZ/IVA groups of the parent study had improvements from parent study baseline at Week 96 in mean sweat chloride concentration (−57.3 (95% CI −61.6– −52.9) and −57.5 (95% CI −62.0– −53.0) $mmol \cdot L^{-1}$), $LCI_{2.5}$ (−1.74 (95% CI −2.09– −1.38) and −2.35 (95% CI −2.72– −1.97) units), FEV_1 %

pred (6.1 (95% CI 2.6–9.7) and 6.9 (95% CI 3.2–10.5) percentage points) and CFQ-R respiratory domain score (6.6 (95% CI 2.5–10.8) and 2.6 (95% CI −1.6–6.8) points).

Conclusions ELX/TEZ/IVA treatment was generally safe and well tolerated, with a safety profile consistent with the parent study and older age groups. After starting ELX/TEZ/IVA, children had robust improvements in sweat chloride concentration and lung function that were maintained through 96 weeks. These results demonstrate the safety and durable efficacy of ELX/TEZ/IVA in this paediatric population.
