# Supplementary material for: Elexacaftor/tezacaftor/ivacaftor in children aged ≥6 years with cystic fibrosis heterozygous for F508del and a minimal function mutation: results from a 96-week open-label extension study
Source: Eur Respir J. 2025 Jul 10;66(1):2402435. doi: 10.1183/13993003.02435-2024 (PMC12256806; doi:10.1183/13993003.02435-2024)
Supplement: Supplementary file 2 [file ERJ-02435-2024.Supplement.pdf]

**Online Data Supplement:**

# Elexacaftor/tezacaftor/ivacaftor in children aged ≥6 years with cystic fibrosis heterozygous for *F508del* and a minimal function mutation: Results from a 96-week open-label extension study

Marcus A. Mall,[1a,b,c*], Claire E. Wainwright,[2*] Julian Legg,[3a,b] Mark Chilvers,[4] Sylvia Gartner,[5] Anna-Maria Dittrich,[6] Florian Stehling,[7] Sarah Conner,[8] Sebastian Grant,[8] Nina Suresh,[8] Tanya G. Weinstock,[8] Jane C. Davies,[9a,b,c] for the VX20-445-119 Study Group

[1a]Department of Pediatric Respiratory Medicine, Immunology and Critical Care Medicine, Charité - Universitätsmedizin Berlin, Berlin, Germany; [1b]German Center for Child and Adolescent Health (DZKJ), partner site, Berlin, Germany; [1c]German Center for Lung Research (DZL), associated partner site Berlin, Berlin, Germany; [2]Queensland Children's Hospital, University of Queensland, Queensland, Australia; [3a]National Institute for Health Research, Southampton respiratory Biomedical Research Centre, University Hospitals Southampton NHS Foundation Trust, Southampton, UK; [3b]Southampton Children's Hospital, University Hospitals Southampton NHS Foundation Trust, Southampton, UK; [4]British Columbia Children's Hospital, University of British Columbia, Vancouver, Canada; [5]Hospital Universitari Vall d'Hebron, Barcelona, Spain; [6] Hannover Medical School, Department for Pediatric Pulmonology, Allergology, and Neonatology, Hannover, Germany and BREATH, German Center for Lung Research (DZL), Hannover, Germany; [7]Children's Hospital, University of Duisburg-Essen, Essen, Germany; [8]Vertex Pharmaceuticals Incorporated, Boston, MA; [9a]National Heart

and Lung Institute, Imperial College London, UK; [9b]Royal Brompton and Harefield Hospitals, part of Guy's and St Thomas' NHS Trust, London, UK; [9c]European CF Society Lung Clearance Index Core Facility, London, UK

**Table of Contents**

**METHODS** ........ **4**

**List of site investigators and coordinators for VX20-445-119** ........ 4

**Inclusion and Exclusion Criteria**: ........ 6

Inclusion Criteria ........ 6

Exclusion Criteria ........ 6

**Multiple-breath Washout** ........ 7

**Figure S1**. Study design ........ 9

**Table S1**. Qualifying minimal function mutations ........ 10

**RESULTS** ........ **12**

**Figure S2**. Mean CFQ-R respiratory domain score by visit ........ 12

**Table S2**. Exposure ........ 13

**Table S3**. Mean CFQ-R respiratory domain score by visit ........ 14

**Table S4**. SAEs occurring in ≥2 children ........ 16

**Table S5**. Threshold analysis of LFT chemistry parameters ........ 17

**Table S6**. Summary of elevated transaminase events ........ 18

**Table S7**. Summary of rash events ........ 21

**Table S8**. Changes in blood pressure ........ 23

**REFERENCES** ........ **25**

## METHODS

### List of site investigators and coordinators for VX20-445-119

The VX20-445-119 investigators included Rossa Brugha, Great Ormond Street Hospital for Children, London, United Kingdom; Olaf Sommerburg, Universitaetsklinikum Heidelberg, Zenter fuer Kinder-und Jugendmedizin, Heidelberg, Germany; Alexander Moeller (Möller), Kinderspital Zuerich, Division of Respiratory Medicine, Zurich, Switzerland; Malena Cohen-Cymberknoh, Hadassah University Hospital Mount Scopus, Jerusalem, Israel; Dario Prais, Schneider Children's Medical Center of Israel, Central District, Israel; Pedro Mondejar, Hospital Virgen de la Arrixaca, Murcia, Spain; Anna-Maria Dittrich, Medizinische Hochschule Hannover, Lower Saxony, Germany; Melinda Solomon, The Hospital for Sick Children, Toronto, ON, Canada; Mark Chilvers, British Columbia Children's Hospital, Vancouver, BC, Canada; Larry Lands, McGill University Health Centre, Glen Site, Montreal Children's Hospital, Montreal, QC, Canada; Philipp Latzin, Inselspital - Universitaetsspital Bern, Medizinbereich Kinder und Jugendliche, Pädiatrische Pneumologie, Bern, Switzerland; Lena Thia, Children and Young Adults Research Unit, Noah's Ark Children's Hospital for Wales, University Hospital of Wales, Heath Park, Wales, United Kingdom; Marcus Mall, Charite Paediatric Pulmonology Department, Berlin, Germany; Olaf Eickmeier, Johann Wolfgang Goethe University, Hesse, Germany; Tacjana Pressler, Juliane Marie Center, Rigshospitalet, Copenhagen, Denmark; Florian Stehling, Kinderklinik III, Abt. fur Pneumologie, North Rhine-Westphalia, Germany; Silke van Koningsbruggen-Rietschel, Universitaetsklinkum Koeln, CF-Studienzentrum, North Rhine-Westphalia, Germany; Lutz Nährlich, Justus-Liebig-Universität Gießen Zentrum für Kinderheilkunde und Jugendmedizin, Hesse, Germany; Jane Davies, Royal Brompton Hospital, London, United Kingdom; Don Urquhart, Royal Hospital for Sick Children, Edinburgh, United

Kingdom; Julian Legg, Southampton General Hospital, Hampshire, United Kingdom; Rebecca Thursfield, Alder Hey Children's NHS Foundation Trust, Liverpool, United Kingdom; Tom Hilliard, University Hospitals Bristol and Weston NHS Foundation Trust, Bristol Royal Hospital, Bristol, United Kingdom; Veronique Houdouin, Hopital Robert Debre, Paris, France; Sophie Ramel, Centre de Perharidy, Brittany, France; Isabelle Sermet-Gaudelus, Hopital Necker, Enfants Malades, Paris, France; Hettie Janssens, Erasmus Medical Center / Sophia Children's Hospital, Rotterdam, Netherlands; Philippe Reix, CHU Lyon - Hopital Femme Mere-Enfant, Auvergne-Rhône-Alpes, France; Stephanie Bui, Groupe Hospitaler Pellegrin, CHU De Bordeaux, Bordeaux cedex, Nouvelle-Aquitaine, France; Gerard Koppelman, Universitair Medisch Centrum Groningen, Gronigen, Netherlands; Silvia Gartner, Hospital Universitari Vall d Hebron, Barcelona, Spain; Hiranjan (Hiran) Selvadurai, The Children’s Hospital at Westmead, Westmead, NSW, Australia; Claire Wainwright, and Queensland Children's Hospital, South Brisbane, QLD, Australia; Barry Clements Telethon Kids Institute, Nedlands, WA, Australia.

**Inclusion and Exclusion Criteria:**

Participants who meet all the following inclusion criteria and none of the exclusion criteria were eligible.

Inclusion Criteria

- Participant (or legally appointed and authorized representative) sign and date an informed consent form and, when appropriate, an assent form.
- Willing and able to comply with scheduled visits, treatment plan, study restrictions, laboratory tests, contraceptive guidelines, and other study procedures.
- Did not withdraw consent from the parent study.
- Meets at least one of the following criteria:
  - Completed study drug treatment in the parent study.
  - Had study drug interruption(s) in the parent study, but did not permanently discontinue study drug, and completed study visits up to the last scheduled visit of the Treatment Period of the parent study.
- Willing to remain on a stable cystic fibrosis treatment regimen through completion of study participation.

Exclusion Criteria

- History of any comorbidity that, in the opinion of the investigator, might confound the results of the study or pose an additional risk in administering study drug to the participant.

- History of drug intolerance in the parent study that would pose an additional risk to the participant in the opinion of the investigator (e.g., participants with a history of allergy or hypersensitivity to the study drug).
- Pregnant and breast-feeding females. Female participants must have a negative pregnancy test at the Day 1 Visit before receiving the first dose of study drug.
- Current participation in an investigational drug trial other than the parent study. Participation in a noninterventional study (including observational studies, registry studies, and studies requiring blood collections without administration of study drug) and screening for another Vertex Pharmaceuticals Incorporated study is permitted.

**Multiple-breath Washout**

Nitrogen ($N_2$) multiple breath washout (MBW) testing was performed with an Exhalyzer-D (Eco Medics) using Spiroware version 3.1.6. The $N_2$-MBW testing was performed in multiple replicates for each visit and the final lung clearance index (LCI) value was calculated from the technically acceptable washout replicates by a central reader. The final LCI value at each visit was the value provided by the LCI vendor based on the replicates. At all visits, all MBW were performed pre-bronchodilator. The MBW was performed before the spirometry assessment.

**Definition of a Minimal Function Variant**

Minimal function (MF) variants were defined in the protocol as the subset of *CFTR* gene variants that meet at least one of the following criteria: (1) no biological plausibility of translated protein (genetic sequence predicts the complete absence of CFTR protein) or (2) in vitro testing supports lack of responsiveness to ivacaftor (IVA) and tezacaftor/ivacaftor (TEZ/IVA). Variants considered to be MF based on in vitro testing met the following criteria in in vitro experiments:

(1) baseline chloride transport that was <10% of wild-type CFTR and (2) an increase in chloride transport of <10% over baseline following the addition of IVA or TEZ/IVA in the assay.

Table S1 lists MF variants detectable by a US Food and Drug Administration–cleared genotyping assay or other method (e.g., sequencing) that was stated in the protocol as meeting the eligibility criteria; the protocol list was not exhaustive, and the protocol instructed investigators to contact the medical monitor regarding other variants that might also meet the eligibility criteria.

**Figure S1.** Study design

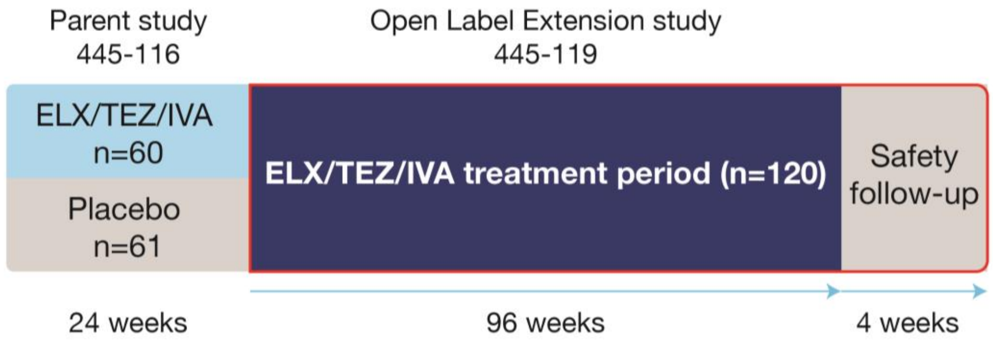


ELX/TEZ/IVA: elexacaftor/ivacaftor/tezacaftor

**Table S1.** Qualifying minimal function variants

| **Variants** | | | | |
|---|---|---|---|---|
| *Q2X* | *L218X* | *Q525X* | *R792X* | *E1104X* |
| *S4X* | *Q220X* | *G542X* | *E822X* | *W1145X* |
| *W19X* | *Y275X* | *G550X* | *W882X* | *R1158X* |
| *G27X* | *C276X* | *Q552X* | *W846X* | *R1162X* |
| *Q39X* | *Q290X* | *R553X* | *Y849X* | *S1196X* |
| *W57X* | *G330X* | *E585X* | *R851X* | *W1204X* |
| *E60X* | *W401X* | *G673X* | *Q890X* | *L1254X* |
| *R75X* | *Q414X* | *Q685X* | *S912X* | *S1255X* |
| *L88X* | *S434X* | *R709X* | *Y913X* | *W1282X* |
| *E92X* | *S466X* | *K710X* | *Q1042X* | *Q1313X* |
| *Q98X* | *S489X* | *Q715X* | *W1089X* | *Q1330X* |
| *Y122X* | *Q493X* | *L732X* | *Y1092X* | *E1371X* |
| *E193X* | *W496X* | *R764X* | *W1098X* | *Q1382X* |
| *W216X* | *C524X* | *R785X* | *R1102X* | *Q1411X* |
| *185+1G→T* | *711+5G→A* | *1717-8G→A* | *2622+1G→A* | *3121-1G→A* |
| *296+1G→A* | *712-1G→T* | *1717-1G→A* | *2790-1G→C* | *3500-2A→G* |
| *296+1G→T* | *1248+1G→A* | *1811+1G→C* | *3040G→C (G970R)* | *3600+2insT* |
| *405+1G→A* | *1249-1G→A* | *1811+1.6kbA→G* | | *3850-1G→A* |
| *405+3A→C* | *1341+1G→A* | *1811+1643G→T* | *3120G→A* | *4005+1G→A* |
| *406-1G→A* | *1525-2A→G* | *1812-1G→A* | *3120+1G→A* | *4374+1G→T* |
| *621+1G→T* | *1525-1G→A* | *1898+1G→A* | *3121-2A→G* | |
| *711+1G→T* | | *1898+1G→C* | | |
| *182delT* | *1119delA* | *1782delA* | *2732insA* | *3791delC* |
| *306insA* | *1138insG* | *1824delA* | *2869insG* | *3821delT* |
| *365-366insT* | *1154insTC* | *1833delT* | *2896insAG* | *3876delA* |
| *394delTT* | *1161delC* | *2043delG* | *2942insT* | *3878delG* |
| *442delA* | *1213delT* | *2143delT* | *2957delT* | *3905insT* |

| | | | | |
|---|---|---|---|---|
| 444delA | 1259insA | 2183AA→G[†] | 3007delG | 4016insT |
| 457TAT→G | 1288insTA | 2184delA | 3028delA | 4021dupT |
| 541delC | 1343delG | 2184insA | 3171delC | 4022insT |
| 574delA | 1471delA | 2307insA | 3171insC | 4040delA |
| 663delT | 1497delGG | 2347delG | 3271delGG | 4279insA |
| 849delG | 1548delG | 2585delT | 3349insT | 4326delTC |
| 935delA | 1609delCA | 2594delGT | 3659delC | |
| 1078delT | 1677delTA | 2711delT | 3737delA | |
| CFTRdele1 | CFTRdele16-17b | 991del5 | | |
| CFTRdele2 | CFTRdele17a,17b | 1461ins4 | | |
| CFTRdele2,3 | CFTRdele17a-18 | 1924del7 | | |
| CFTRdele2-4 | CFTRdele19 | 2055del9→A | | |
| CFTRdele3-10,14b-16 | CFTRdele19-21 | 2105-2117del13insAGAAA | | |
| CFTRdele4-7 | CFTRdele21 | 2372del8 | | |
| CFTRdele4-11 | CFTRdele22-24 | 2721del11 | | |
| CFTR50kbdel | CFTRdele22,23 | 2991del32 | | |
| CFTRdup6b-10 | 124del23bp | 3121-977_3499+248del2515 | | |
| CFTRdele11 | 306delTAGA | 3667ins4 | | |
| CFTRdele13,14a | 602del14 | 4010del4 | | |
| CFTRdele14b-17b | 852del22 | 4209TGTT→AA | | |
| A46D | V520F | Y569D | N1303K | |
| G85E | A559T | L1065P | | |
| R347P | R560T | R1066C | | |
| L467P | R560S | L1077P | | |
| I507del | A561E | M1101K | | |

*This list of qualifying variants was developed based on CFTR2.org (1).

[†]Also known as *2183delAA→G*.

# RESULTS

**Figure S2.** Mean CFQ-R respiratory domain score by visit

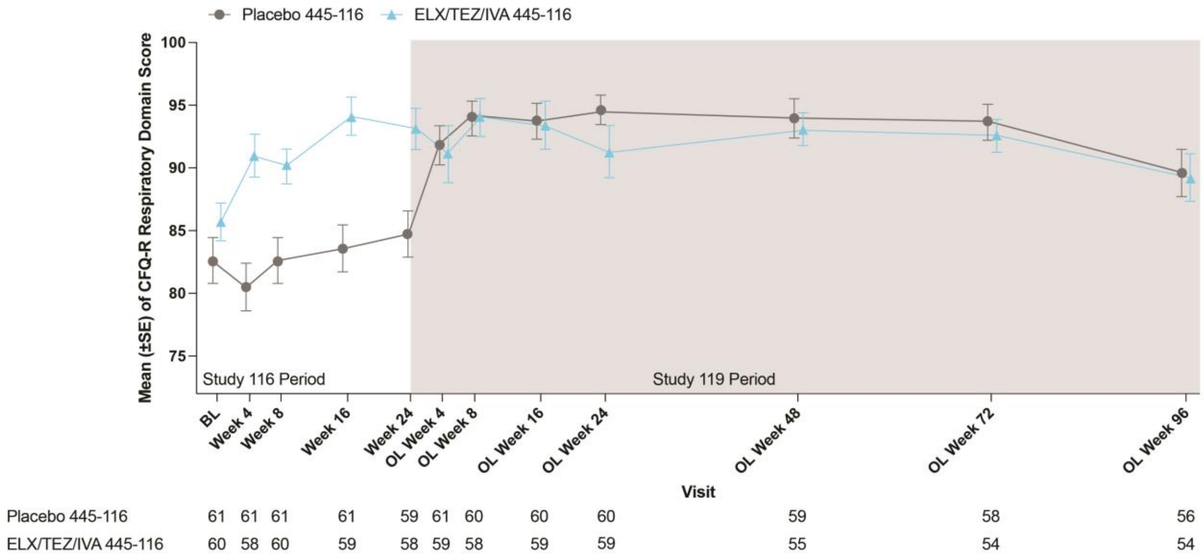


BL: baseline; CFQ-R: Cystic Fibrosis Questionnaire-Revised (Child's version); ELX/TEZ/IVA: elexacaftor/ivacaftor/tezacaftor; OL: open label; SE: standard error

The values below the x-axis are n values.

All children were treated with ELX/TEZ/IVA in the OL extension study.

**Table S2.** Exposure

| Category | Any ELX/TEZ/IVA N = 120 |
|---|---|
| Exposure duration (weeks) | |
| n | 120 |
| Mean (SD) | 92.9 (12.4) |
| Median | 96.0 |
| Exposure duration by interval, n (%) | |
| ≤24 weeks | 1 (0.8) |
| >24 to ≤48 weeks | 1 (0.8) |
| >48 to ≤72 weeks | 5 (4.2) |
| >72 to ≤96 weeks | 61 (50.8) |
| >96 weeks | 52 (43.3) |

ELX: elexacaftor; IVA: ivacaftor; n: size of subsample; N: total sample size; SD: standard deviation; TEZ: tezacaftor

Duration of study drug exposure (weeks) = (last dose date of study drug in the open label extension study - first dose date of study drug in the open label extension study + 1)/7, regardless of study drug interruption.

**Table S3.** Mean CFQ-R respiratory domain score by visit

| | **Parent Study→Open Label Extension** | | | | **445-106→445-107 Part A** | |
|---|---|---|---|---|---|---|
| | **Placebo→ ELX/TEZ/IVA N=61→61** | | **ELX/TEZ/IVA→ ELX/TEZ/IVA N=60→59** | | **ELX/TEZ/IVA→ ELX/TEZ/IVA N=66→64** | |
| | **n** | **Mean (SE)** | **n** | **Mean (SE)** | **n** | **Mean (SE)** |
| Parent Study Baseline | 61 | 82.7 (1.8) | 60 | 85.7 (1.5) | 65 | 80.3 (1.9) |
| Average Through Week 24 | 61 | 82.9 (1.5) | 60 | 91.8 (1.3) | 66 | 87.1 (1.2) |
| **Open Label Extension** | | | | | **445-107 Part A** | |
| Week 4 | 61 | 91.8 (1.5) | 59 | 91.1 (2.3) | -- | -- |
| Week 8 | 60 | 94.2 (1.2) | 58 | 94.0 (1.5) | 33 | 93.7 (1.6) |
| Week 16 | 60 | 93.8 (1.4) | 59 | 93.4 (1.9) | -- | -- |
| Week 24 | 60 | 94.6 (1.1) | 59 | 91.2 (2.1) | 61 | 92.5 (1.3) |
| Week 48 | 59 | 93.9 (1.5) | 55 | 93.0 (1.3) | 63 | 92.5 (1.3) |
| Week 72 | 58 | 93.7 (1.4) | 54 | 92.6 (1.3) | 58 | 92.2 (1.2) |
| Week 96 | 56 | 89.6 (1.9) | 54 | 89.2 (1.9) | 60 | 93.3 (1.0) |
| 95% CI for Open Label Week 96 | | (85.8, 93.3) | | (85.5, 93.0) | | (91.3, 95.3) |

CFQ-R: Cystic Fibrosis Questionnaire-Revised (Child's version); CI: confidence interval; ELX: elexacaftor; IVA: ivacaftor; n: size of subsample; N: total sample size; SE: standard error; TEZ: tezacaftor

Descriptive SEs and 95% CIs are from ad-hoc analyses.

**Table S4.** SAEs occurring in ≥2 children

| | ELX/TEZ/IVA N = 120 | |
|---|---|---|
| **Category** | **n (%)** | **Events/100PY** |
| SAEs Occurring in ≥2 Children (PT) | | |
| Children with any SAEs | 13 (10.8) | 7.30 |
| Infective PEx of CF | 2 (1.7) | 0.86 |

CF: cystic fibrosis; ELX/TEZ/IVA: elexacaftor/ivacaftor/tezacaftor; n: size of subsample; N: total sample size; PEx: pulmonary exacerbation; PT: Preferred Term; PY: patient-years; SAE: serious adverse event

MedDRA version 25.1 was used for Study 119 and MedDRA 24.0 for Study 116.

A child with multiple events within a category (Overall or PT) was counted only once in that category.

**Table S5.** Threshold analysis of LFT chemistry parameters

| | Parent Study 116 | | Open-Label Extension Study 119 |
|---|---|---|---|
| | **Placebo in Study 116 N = 61** | **ELX/TEZ/IVA in Study 116 N = 60** | **Any ELX/TEZ/IVA in Study 119 N = 120** |
| Total, N1 | 61 | 59 | 120 |
| ALT (U/L) or AST (U/L), n (%) | | | |
| >3 × ULN | 3 (4.9) | 8 (13.6) | 11 (9.2) |
| >5 × ULN | 1 (1.6) | 3 (5.1) | 6 (5.0) |
| >8 × ULN | 0 | 1 (1.7) | 0 |
| Total bilirubin (µmol/L), n (%) | | | |
| >ULN to ≤1.5 × ULN | 1 (1.6) | 2 (3.4) | 6 (5.0) |
| >1.5 × to ≤2 × ULN | 0 | 0 (0) | 3 (2.5) |
| >2 × to ≤3 × ULN | 0 | 1 (1.7) | 0 |
| >3 × ULN | 0 | 0 | 1 (0.8) |

ALT: alanine transaminase; AST: aspartate transaminase; ELX/TEZ/IVA: elexacaftor/ivacaftor/tezacaftor; LFT: liver function test; n: size of subsample; N: total sample size; N1: The number of participants with at least one nonmissing measurement during the treatment-emergent period; ULN: upper limit of normal

ELX/TEZ/IVA safety baseline was defined as the most recent non-missing measurement collected before the first dose of study drug in the parent study or the first dose of study drug in the open-label extension study.

A child was counted in all applicable post-ELX/TEZ/IVA-safety-baseline categories based on the worst assessment during the treatment-emergent period; percentage is n/N1.

**Table S6.** Summary of elevated transaminase events

| | Parent Study 116 | | | | Open-Label Extension Study 119 | |
|---|---|---|---|---|---|---|
| | Placebo in Study 116 N = 61 | | ELX/TEZ/IVA in Study 116 N = 60 | | Any ELX/TEZ/IVA N = 120 | |
| **Patients, n (%)** | **n (%)** | **Events/100PY** | **n (%)** | **Events/100PY** | **n (%)** | **Events/100PY** |
| Any Elevated Transaminase events | 3 (4.9) | 19.52 | 6 (10.0) | 40.17 | 11 (9.2) | 9.88 |
| Alanine aminotransferase increased | 3 (4.9) | 13.01 | 5 (8.3) | 30.13 | 11 (9.2) | 6.44 |
| Aspartate aminotransferase increased | 1 (1.6) | 6.51 | 3 (5.0) | 10.04 | 6 (5.0) | 3.44 |
| AEs by maximum severity | | | | | | |
| Grade 1/Mild | 2 (3.3) | -- | 3 (5.0) | -- | 10 (8.3) | -- |
| Grade 2/Moderate | 1 (1.6) | -- | 3 (5.0) | -- | 1 (0.8) | -- |
| Grade 3/Severe | 0 | -- | 0 | -- | 0 | -- |
| Grade 4/Life-threatening | 0 | -- | 0 | -- | 0 | -- |
| AEs leading to treatment discontinuation | 0 | 0 | 0 | 0 | 0 | 0 |
| AEs leading to treatment interruption | 0 | 0 | 4 (6.7) | 20.08 | 2 (1.7) | 1.29 |

| | Parent Study 116 | | | | Open-Label Extension Study 119 | |
|---|---|---|---|---|---|---|
| | Placebo in Study 116 N = 61 | | ELX/TEZ/IVA in Study 116 N = 60 | | Any ELX/TEZ/IVA N = 120 | |
| Patients, n (%) | n (%) | Events/100PY | n (%) | Events/100PY | n (%) | Events/100PY |
| Serious AEs | 0 | 0 | 0 | 0 | 0 | 0 |
| Duration of events (days) | | | | | | |
| Number of events | 6 | -- | 12 | -- | 23 | -- |
| Number of events with duration | 5 | -- | 11 | -- | 18 | -- |
| Mean (SD) | 10.4 (8.5) | -- | 25.7 (15.1) | -- | 95.6 (106.3) | -- |
| Median | 7.0 | -- | 23.0 | -- | 54.0 | -- |
| Min, max | 2, 20 | -- | 4, 58 | -- | 2, 339 | -- |

AE: adverse event; ELX/TEZ/IVA: elexacaftor/ivacaftor/tezacaftor; n: size of subsample; N: total sample size; OLE: open-label extension; PY: patient-years; SD: standard deviation

Elevated transaminase events were coded using MedDRA version 25.1 (Study 119); MedDRA 24.0 for Study 116.

For number of events, a child with multiple events within a category was counted multiple times in that category.

For number of children with events, a child with multiple events within a category was counted only once in that category.

For number of children with related (serious) events, events with relationship of related, possibly related, and missing were counted.

Duration was only calculated for the events with complete start and end dates.

Children with only one event which had missing severity were summarized in the “Missing” category.

**Table S7.** Summary of rash events

| | Parent Study 116 | | | | Open-Label Extension Study 119 | |
|---|---|---|---|---|---|---|
| | **Placebo in Study 116 N = 61** | | **ELX/TEZ/IVA in Study 116 N = 60** | | **Any ELX/TEZ/IVA in Study 119 N = 120** | |
| **Patients, n (%)** | **n (%)** | **Events/100PY** | **n (%)** | **Events/100PY** | **n (%)** | **Events/100PY** |
| Any events | 3 (4.9) | 9.76 | 8 (13.3) | 30.13 | 13 (10.8) | 6.87 |
| Male | 2 (7.7) | 15.29 | 4 (16.0) | 31.67 | 3 (5.9) | 3.06 |
| Female | 1 (2.9) | 5.66 | 4 (11.4) | 29.00 | 10 (14.5) | 9.65 |
| Events by maximum severity | | | | | | |
| Grade 1/Mild | 3 (4.9) | -- | 3 (5.0) | -- | 10 (8.3) | -- |
| Grade 2/Moderate | 0 | -- | 3 (5.0) | -- | 3 (2.5) | -- |
| Grade 3/Severe | 0 | -- | 2 (3.3) | -- | 0 | -- |
| Grade 4/Life threatening | 0 | -- | 0 | -- | 0 | -- |
| Events leading to treatment discontinuation | 0 | 0 | 1 (1.7) | 3.35 | 0 | 0 |
| Events leading to treatment interruption | 0 | 0 | 2 (3.3) | 6.69 | 1 (0.8) | 0.43 |
| Serious events | 0 | 0 | 1 (1.7) | 3.35 | 0 | 0 |
| Time-to-onset of first event (Days) | | | | | | |
| Mean (SD) | 71.7 (55.3) | -- | 40.6 (58.6) | -- | 121.8 (216.3) | -- |
| Median | 47.0 | -- | 9.5 | -- | 45.0 | -- |
| Min, max | 33, 135 | -- | 8, 139 | -- | 2, 656 | -- |
| Duration of events (Days) | | | | | | |
| Number of events | 3 | -- | 9 | -- | 16 | -- |

| | Parent Study 116 | | | | Open-Label Extension Study 119 | |
|---|---|---|---|---|---|---|
| | Placebo in Study 116 N = 61 | | ELX/TEZ/IVA in Study 116 N = 60 | | Any ELX/TEZ/IVA in Study 119 N = 120 | |
| **Patients, n (%)** | **n (%)** | **Events/100PY** | **n (%)** | **Events/100PY** | **n (%)** | **Events/100PY** |
| Number of events with duration | 3 | -- | 9 | -- | 12 | -- |
| Mean (SD) | 26.3 (37.2) | -- | 13.9 (13.2) | -- | 16.9 (30.7) | -- |
| Median | 9.0 | -- | 9.0 | -- | 5.5 | -- |
| Min, max | 1, 69 | -- | 2, 45 | -- | 1, 105 | -- |

ELX/TEZ/IVA: elexacaftor/ivacaftor/tezacaftor; n: size of subsample; N: total sample size; PY: patient-years; SD: standard deviation

**Table S8.** Changes in blood pressure

| | Parent Study 116 | | Open-Label Extension Study 119 |
|---|---|---|---|
| | **Placebo in Study 116 N=61** | **ELX/TEZ/IVA in Study 116 N=60** | **Any ELX/TEZ/IVA in Study 119 N=120** |
| Systolic blood pressure | | | |
| Baseline, mean, mm Hg* (SD) | 102.2 (9.7) | 103.7 (10.0) | 103.3 (9.4) |
| Change from baseline, mean, mm Hg* (SD) | | | |
| At Day 15 | 0.9 (9.7) | 0.1 (9.9) | NA |
| At Week 4 | 0.0 (9.7) | 0.3 (10.2) | 0.3 (8.9) |
| At Week 8 | 1.3 (11.2) | 2.6 (9.5) | 0.5 (10.9) |
| At Week 16 | 2.6 (10.0) | 1.7 (10.7) | 1.0 (7.9) |
| At Week 24 | 1.0 (8.4) | 1.7 (9.1) | 1.0 (9.5) |
| At Week 36 | NA | NA | 2.0 (9.5) |
| At Week 48 | NA | NA | 2.8 (9.1) |
| At Week 72 | NA | NA | 2.9 (9.9) |
| At Week 96 | NA | NA | 3.2 (10.1) |
| Diastolic blood pressure | | | |
| Baseline, mean, mm Hg* (SD) | 61.7 (7.2) | 65.3 (10.3) | 64.0 (8.6) |

| Change from baseline, mean, mm Hg* (SD) | | | |
|---|---|---|---|
| At Day 15 | 0.3 (8.0) | −2.1 (8.9) | NA |
| At Week 4 | −0.3 (7.4) | −1.5 (8.5) | -0.9 (9.7) |
| At Week 8 | 1.3 (9.0) | 1.1 (12.0) | -1.0 (9.3) |
| At Week 16 | 0.2 (7.3) | −0.4 (11.9) | -1.2 (8.7) |
| At Week 24 | 1.2 (8.6) | −0.6 (8.8) | -0.9 (8.9) |
| At Week 36 | NA | NA | -0.6 (9.0) |
| At Week 48 | NA | NA | -0.1 (10.0) |
| At Week 72 | NA | NA | 0.2 (8.8) |
| At Week 96 | NA | NA | 1.0 (9.0) |
| Adverse events of blood pressure elevation, n (%) | 0 | 0 | 3 (2.5) |
| Serious AEs, n (%) | 0 | 0 | 0 |
| AEs leading to discontinuation, n (%) | 0 | 0 | 0 |
| AEs leading to interruption, n (%) | 0 | 0 | 0 |

AE: adverse event; ELX/TEZ/IVA: elexacaftor/ivacaftor/tezacaftor; NA: not available; SD: standard deviation

*Baseline is defined as the most recent nonmissing measurement before the first dose of study drug in the treatment period.

**REFERENCES**

1. The clinical and functional translation of CFTR (CFTR2). 2016 [cited 2016 Feb 15]. Available from: https://www.cftr2.org/.
